# Epicardial mapping and ablation for ventricular arrhythmias in experienced center without onsite cardiac surgery

**DOI:** 10.21542/gcsp.2021.3

**Published:** 2021-04-30

**Authors:** Shaojie Chen, K.R. Julian Chun, Stefano Bordignon, Shota Tohoku, Boris Schmidt

**Affiliations:** Cardioangiologisches Centrum Bethanien (CCB), Kardiologie, Medizinische Klinik III, Agaplesion Markus Krankenhaus, Akademisches Lehrkrankenhaus der Goethe-Universität Frankfurt am Main, Frankfurt am Main, Germany; Die Sektion Medizin, Universität zu Lübeck, Lübeck, Germany

## Abstract

**Objective:** Epicardial access is sometimes required to effectively treat ventricular arrhythmias, but it can be associated with increased risk of procedural complications needing surgical intervention. The present study aimed to evaluate the feasibility and safety of epicardial mapping/ablation in experienced center without onsite cardiac surgery.

**Methods:** Patients who had drug-refractory, recurrent ventricular arrhythmias were scheduled for catheter ablation. All operators (SC, JC, SB, BS) had at least fifty pericardial puncture experiences. Epicardial puncture and perioperative anticoagulation were carried out based on institutional protocol. Phrenic nerve was mapped by 3-D mapping system. Coronary anatomy was delineated by coronary angiography.

**Results:** A total of 44 patients (63.3 years, male 86.4%) received epicardial access. Of them 7 (15.9%) were scheduled for PVC ablation, 37 (84.1%) for VT ablation (ICM: 25%, NICM: 59.1%). Mean LVEF was 41.3%. Acute ablation success rate was 35 (79.5%). Procedural adverse events included: pericardial effusion occurred in 3 (6.8%) patients who all well treated with pericardial drainage; and pericardial tamponade in 1 (2.3%) patient requiring transfer to surgical intervention. No death, stroke, phrenic nerves palsy, or coronary artery injury were observed. Median hospitalization was 4 (3–6) days. Univariable analysis and ROC curve showed that patients’ age was a significant predictor of epicardial procedural complication (area under curve (AUC): 0.813, *P* = 0.041).

**Conclusions:** Guided by a tailored procedural protocol, the majority of the epicardial access related complications can be treated conservatively without needing onsite surgery. Older age is a risk factor associated with epicardial access related complications.

## Introduction

Ventricular arrhythmias (VA), particularly ventricular tachycardia (VT), is one of the most challenging medical conditions. Antiarrhythmic drugs (AADs) usually have limited effectiveness and are poorly tolerated.^1,2^ Catheter ablation has been increasingly utilized to better treat patients with VAs. The majority of the ablations are performed endocardially, while epicardial mapping and ablation is sometimes required.^3,4^

Access to the pericardial space is usually achieved by the percutaneous approach described by Sosa et al,^5^ and the major risk of pericardial access is pericardial bleeding.^6,7^ Because of these important complications, current international guidelines^8^ recommend onsite surgical support. Considering that the epicardial access related complications are preventable, and the majority of the complications can be managed conservatively, we hypothesized that it is feasible to carry out epicardial mapping/ablation for indicated patients without onsite surgical support in experienced centers.

## Method

### Patient population

All clinical management, procedure, and data collection comply with the Declaration of Helsinki. The ablation procedures were performed in our center without conventional onsite surgical backup. Indications for epicardial mapping and ablation were as follows:

1. Failed endocardial ablation.
2. ECG morphology of VA/VT suggestive of epicardial origin.
3. Underlying cardiac diseases or etiology (i.e., ARVC, DCM) suggestive of epicardial substrate.

### Pericardial access

The epicardium was accessed percutaneously by using the technique described by Sosa et al.^5^ All surgeons had at least fifty pericardial puncture experiences. We performed subxyphoid pericardial puncture with a 17 G-Tuohy needle. The patient was under deep sedation by using boluses of midazolam and a continuous infusion of propofol (1%) under continuous monitoring of electrocardiogram, direct arterial blood pressure and O_2_ saturation. The puncture direction was guided by fluoroscopic imaging in different projections (anterior-posterior view, RAO 30°, LAO 40°, and left lateral view if needed). The decision of entering the pericardium with an anterior or posterior approach was based on the potential origin of the VA/VT or the substrate. Small contrast injection via the needle helped to identify the tenting of the pericardium. Once the pericardium space was reached, immediate advancing a long guidewire assisted to obtain the pericardium access. Fluoroscopy was used to confirm the guidewire crossing around the pericardium without entrance into the cardiac chambers. A steerable sheath was then advanced over the guide-wire; thereafter a mapping/ablation catheter was advanced to the pericardium via the steerable sheath to perform mapping and ablation.

Perioperative oral anticoagulants (OACs) were stopped the day of the procedure; heparin was administered individually before the procedure. Phrenic nerve was mapped by 3-D mapping system. Coronary anatomy was mapped by selective coronary angiography. Epicardial drainage was deployed in patients who had hemopericardium post-procedure. Transthoracic echocardiography (TTE) was routinely performed peri-procedural and in-hospital follow-up.

### Management of anticoagulation during procedure

**Figure 1.**
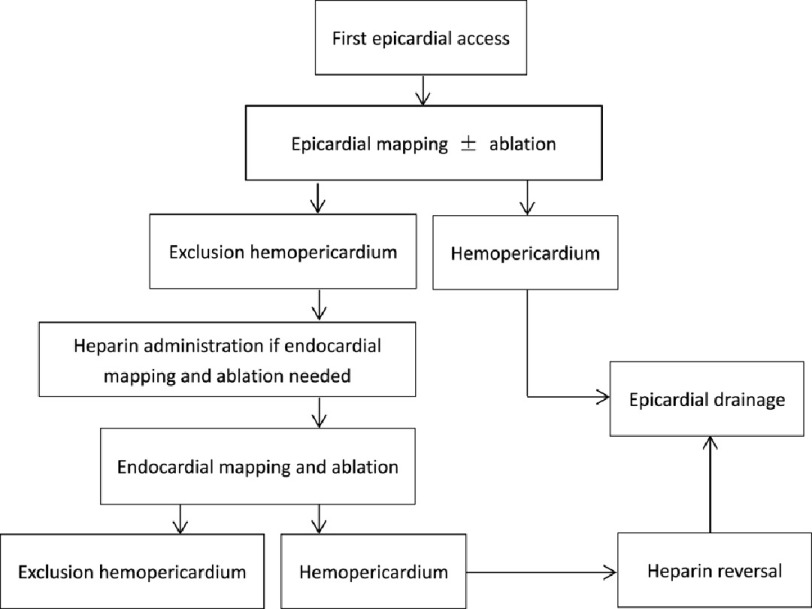
Epicardial/endocardial access, and anticoagulation flowchart.

In principle, pericardial access was obtained prior to systemic anticoagulation or after reversal of systemic anticoagulation. Continuous monitoring for pericardial bleeding was performed throughout the procedure. Anticoagulation was administered for further endocardial mapping and ablation if pericardial access was confirmed without active bleeding. The target active clotting time (ACT) for endocardial mapping and ablation was 300. The detailed flowchart protocol is summarized in Figure 1.

### Electrophysiological study and ablation

**Figure 2.**
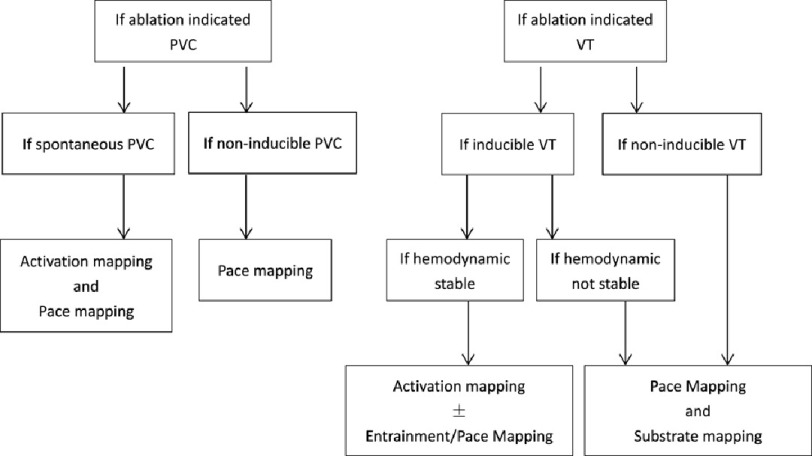
Mapping and ablation strategy. (PVC: premature ventricular contraction, VT: ventricular tachycardia).

Epicardial mapping was principally performed before endocardial mapping. A 3-D electroanatomical epicardial map (CARTO, Biosense Webster) was created by using an irrigated-tip radiofrequency (RF) catheter (ThermoCool, SF/STSF, Biosense Webster). Epicardial ablation was performed: (1) if local early activation (goal >−30 ms prior to begin of the QRS) was identified during clinical VA/VT, and local unipolar recording with qS complex; or (2) if epicardial pace mapping demonstrated >10 out of 12 match for a target VA/VT, or (3) if local abnormal ventricular activity (LAVA) potentials typically late potentials were seen within epicardial scar defined as low voltage (0.5–1.0 mv under sinus rhythm) area. The detailed mapping and ablation strategy was summarized in Figure 2.

Radiofrequency ablation (30–40 W, 60 seconds per site) was performed by using an irrigated (ThermoCool, SF/STSF Biosense Webster) at a flow rate of 8–15 ml/min with temperature limit of 40 °C.

### Complications prevention and management

The epicardial sheath was continuously aspirated; pericardial bleeding was monitored throughout the procedure. Coronary angiographies were performed to outline the major epicardial vessels relative to the potential region of ablation. Epicardial ablation sites should be at least 5 mm away from the coronary vessel. The course of the phrenic nerve was identified by high output pacing (20 mA, 2 ms pulse width) prior to epicardial ablation.

At the end of the procedure all pericardial residual fluid was aspirated. After the aspiration, for patients who had >20 ml additional effusion, epicardial drainage was retained; if no further evidence of pericardial effusion or bleeding during one day observation, the epicardial drainage would be removed at the second day after the procedure, and preventive antibiotic therapy was administered until removal of the epicardial drainage. If continuous pericardial bleeding (aspirated volume >1000 ml) despite of conservative management, the patient would be transferred to the surgical department in the university hospital within 20–30 min’s driving time (predefined transfer agreement and protocol). During the transfer, the patient was under continuous monitoring accompanied by experienced intensive care physicians, medications and blood transfusions for emergency were prepared on board.

### Procedural outcome assessment

For patients with VT, after ablation programmed stimulation was repeated with the same protocol as baseline. As institutional standard, the basic stimulation drive was 510 ms and 440 ms, combined with 1–3 extra beat stimulation until ventricular refractory, the programmed stimulation were repeated at right ventricular apex and outflow tract. Repeated stimulation was performed after discontinuation of the sedation. Procedural success was defined as non-inducibility of any VT. Partial success was defined as inducibility of nonclinical VT. For idiopathic premature ventricular contraction (PVC), procedural success was defined as the absence of PVC during 30 min waiting period after the last ablation under discontinuation of the sedation.

### Clinical follow-up

Outpatient clinic follow-up was scheduled in every six-month interval, including clinical assessment, Holter monitoring, device interrogation, and transthoracic echocardiography. Patients who had any symptoms suggesting of arrhythmia recurrence were contacted for clinical evaluation.

### Statistical analysis

Continuous variables were expressed as mean and standard deviation (SD) and compared by using Student *t*-test. Categorical variables were described as numbers and percentages and compared by using the Chi-square test or the Fisher exact test. *P* value < 0.05 was considered statistically significant. The statistical analyses were performed using the SPSS statistical package version 17.0 (SPSS Inc. Chicago).

## Results

### Patients

Data from 44 consecutive patients who underwent epicardial mapping and/or ablation were collected. Mean age was 63.3 ± 13.2 years, male patients were 86.4%. Mean left ventricular ejection fraction (LVEF) was 41.3 ± 12.9%. The baseline characteristics were summarized in Table 1.

**Table 1:** Demographic characteristics.

Sample size, N	44
Age, years	63.3 ± 13.2
Male, n (%)	38 (86.4%)
BMI, (kg/m^2^)	28.5 ± 4
Hypertension, n (%)	19 (43.2%)
Diabetes II, n (%)	8 (18.2%)
CAD, n (%)	15 (34.1%)
Heart failure, n (%)	42 (95.5%)
NYHA classification	III (II–III)
CKD, n (%)	11 (25%)
AF, n (%)	16 (36.4%)
Type of VAs	
*PVC, n (%)	7 (15.9%)
*VT, n (%)	37 (84.1%)
ICM, n (%)	11 (25%)
DCM, n (%)	18 (40.9%)
HCM, n (%)	3 (6.8%)
ARVC, n (%)	5 (11.4%)
LVEDD, mm	58 ± 7.9
LVEF	41.3 ± 12.9%
Beta-Blocker, n (%)	37 (84.1%)
Amiodarone, n (%)	30 (68.2%)
ACEI/ARB, n (%)	22 (50%)
Spirolactone, n (%)	15 (34.1%)
OACs, n (%)	16 (36.4%)
ICD, or CRT-D, n (%)	37 (84.1%)

**Notes.**

BMI body mass index CAD coronary artery disease NYHA New York Heart Association CKD chronic kidney disease AF atrial fibrillation VA ventricular arrhythmia PVC premature ventricular contraction VT ventricular tachycardia ICM ischemic cardiomyopathy DCM dilated cardiomyopathy HCM hypertrophic cardiomyopathy ARVC arrhythmogenic right ventricular cardiomyopathy LVEDD left ventricular end diastolic diameter LVEF left ventricular ejection fraction ACEI angiotensin-converting-enzyme inhibitor ARB angiotensin II receptor blocker OAC oral anticoagulation

### Indications for epicardial mapping/ablation

**Figure 3.**
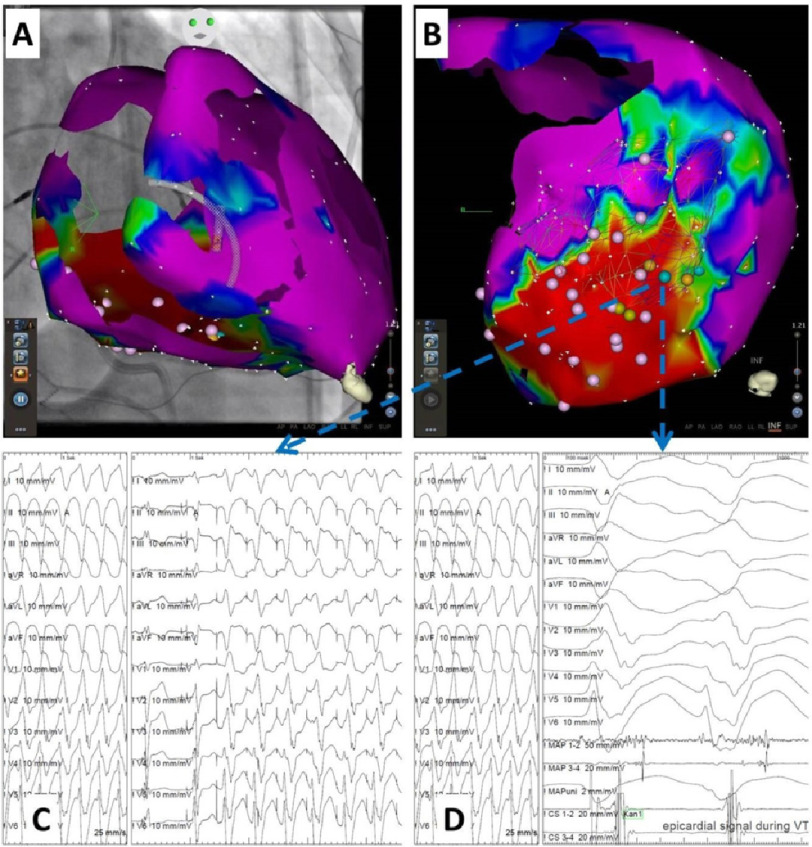
Epicardial mapping and ablation in ICM.

Seven (15.9%) patients had frequent recurrent premature ventricular contraction (PVC) despite of endocardial ablation. Thirty-seven (84.1%) patients had recurrent ventricular tachycardia, of them eleven (25%) patients had ischemic cardiomyopathy (ICM), eighteen (40.9%) patients had dilated cardiomyopathy, three (6.8%) patients had hypertrophic cardiomyopathy, and five (11.4%) patients had arrhythmogenic right ventricular cardiomyopathy (ARVC). Figure 3 shows an example of epicardial mapping/ablation in a patient with ICM.

### Procedural data

Pericardial access was successfully deployed in all 44 (100%) patients. Thirty-seven (84.1%) patients received both epicardial mapping and epicardial ablation. Seven (15.9%) patients received only epicardial mapping without ablation, of them in two patients, the pericardial space could not be fully accessed due to adhesion. Of the remaining five patients, no epicardial LAVA was found instead endocardial ablation targets were found. The mean number of VT was 2.4 ± 1.5. Procedural success was achieved in 35 (79.5%) patients. The procedural data are summarized in Table 2.

**Table 2:** Procedural data.

Sample size, N	44
Epicardial mapping and ablation, n (%)	37 (84.1%)
Epicardial mapping only, n (%)	7 (15.9%)
Pericardial adhesion, n (%)	2 (4.5%)
VAs origin from LV, n (%)	33 (75%)
VAs origin from RV, n (%)	11 (25%)
Number of VTs, n	2.4 ± 1.5
Cycle length of clinical VT, ms	310 (276–365)
Procedural success	35 (79.5%)
Procedural time. min	180 (120–200)
Fluoroscopic time. min	18.2 (10.5–22.9)
	
Overall procedural adverse events	4 (9.1%)
Pericardial effusion	3 (6.8%)
Pericardial bleeding/tamponade	1 (2.3%)
Other major bleeding	0
Coronary injury	0
Stroke	0
PNP	0
Other organ damage	0
Death	0
Complication needing transfer for surgery	1 (2.3%)
	
Hospital stay. days	4 (3–6)
12 months VT recurrence	15 (40.1%)

**Notes.**

VA ventricular arrhythmia LV left ventricle RV right ventricle VT ventricular tachycardia PNP phrenic nerve palsy

### Adverse events

As shown in Table 2, overall procedural adverse events occurred in four (9.1%) patients. No death or other serious complications occurred. Three patients had pericardial effusion which were managed by epicardial drainage and conservative treatment, the mean time of drainage was 1.1 ± 0.2 days, and the mean aspirated effusion volume was 16 ± 4 ml.

One patient had pericardial tamponade (aspirated bleeding volume 1000 ml, hemoglobin dropped from 16 g/dl to 13 g/dl) one day after the procedure; within the same day, the patient was transferred to surgical center due to recurrent pericardial bleeding despite of internal treatment. During the thoracotomy surgery, further 300 ml pericardial bleeding was aspirated, blood clot was removed, and one epicardial bleeding spot was found and repaired. The operation was successful, and the patient was discharged ten days after the surgery.

### Univariable comparison for patients with or without hemopericardium

The univariable comparison showed that, patients with hemopericardium was significantly older (77.8 ± 12.8 years vs. 61.8 ± 12.5 years, *P* = 0.019) as compared to patients without hemopericardium. There were no significant differences regarding other baseline characteristics and procedural data. Patients with hemopericardium needed significantly longer hospital stay (16.3 ± 10.4 days vs. 4.3 ± 2.3 days, *P* < 0.01) as compared to patients without hemopericardium. The detailed univariable comparisons were presented in Table 3.

**Table 3:** Univariable comparison for patients with or without hemopericardium.

Variables	Patients had Hemopericardium, *N* = 4	Patients without Hemopericardium, *N* = 40	*P* value
Age, years	77.8 ± 12.8	61.8 ± 12.5	**0.019**
BMI, kg/m^2^	28.0 ± 5.2	28.6 ± 4.0	0.822
Male gender, n (%)	3 (75.0%)	35 (87.5%)	0.487
Hypertension, n (%)	0 (0%)	19 (47.4%)	0.067
Diabetes, n (%)	0 (0%)	8 (20.0%)	0.323
CAD, n (%)	0 (0%)	15 (37.5%)	0.131
HF, n (%)	4 (100%)	38 (95.0%)	0.647
NYHA classification,	3.0 ± 0	2.4 ± 0.8	0.181
CKD, n (%)	2 (50.0%)	9 (22.5%)	0.226
CKD classification,	1.5 ± 1.7	0.6 ± 1.1	0.114
AF, n (%)	3 (75.0%)	13 (32.5%)	0.092
ICM, n (%)	0 (0%)	11 (27.5%)	0.226
DCM, n (%)	3 (75.0%)	15 (37.5%)	0.146
HCM, n (%)	1 (25.0%)	2 (5.0%)	0.130
ARVC, n (%)	0 (0%)	5 (12.5%)	0.453
Vavular heart disease, n (%)	0 (0%)	1 (2.5%)	0.749
Beta-Blocker, n (%)	4 (100%)	33 (82.5%)	0.362
Amiodarone, n (%)	4 (100%)	26 (65.0%)	0.152
ACEI/ARB, n (%)	3 (75%)	19 (47.5%)	0.294
Spirolactone, n (%)	2 (50%)	13 (32.5%)	0.481
OACs, n (%)	3 (75.0%)	13 (32.5%)	0.129
VA origin form LV, n (%)	4 (100%)	29 (76.3%)	0.272
VT cycle length, ms	330.0 ± 60.0	319.4 ± 54.1	0.723
LVEED, mm	58.8 ± 9.1	57.9 ± 7.9	0.849
LVEF, %	37.5 ± 13.2	41.8 ± 12.9	0.539
Acute success, n (%)	3 (75.0%)	32 (80.0%)	0.267
Procedure time, min	207.5 ± 15.0	157.8 ± 52.7	0.07
Fluoroscopic time, min	21.2 ± 1.8	18.2 ± 8.8	0.508
Hospital stay, day	16.3 ± 10.4	4.3 ± 2.3	<0.01
In-hospital VT recurrence, n (%)	1 (25.0%)	5 (15.1%)	0.62
12-month VT recurrence, n (%)	3 (75.0%)	12 (36.4%)	0.17

**Notes.**

BMI body mass index CAD coronary artery disease HF heart failure NYHA New York Heart Association CKD chronic kidney disease ICM ischemic cardiomyopathy DCM dilated cardiomyopathy HCM hypertrophic cardiomyopathy ARVC arrhythmogenic right ventricular cardiomyopathy ACEI angiotensin-converting-enzyme inhibitor ARB angiotensin II receptor blocker OAC oral anticoagulation VA ventricular arrhythmia VT ventricular tachycardia

### Receiver Operator Characteristic (ROC) curve

**Figure 4.**
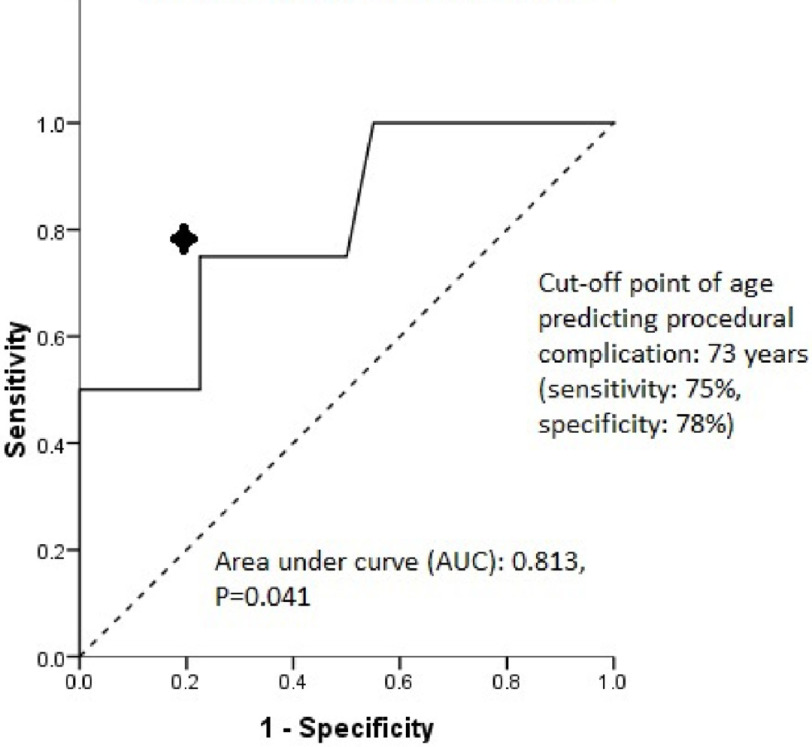
ROC curve age against procedural complication.

As demonstrated in Figure 4, The Receiver Operator Characteristic (ROC) curve analysis using age against procedural complication showed that the patients’ age was a significant predictor of epicardial procedural complication (area under curve (AUC): 0.813, *P* = 0.041). In this cohort, the cut-off point of age predicting procedural complication was 73 years (sensitivity: 75%, specificity: 78%).

## Discussion

Management of ventricular arrhythmias particularly ventricular tachycardia represents a challenge in clinical practice. For patients with ventricular arrhythmias in the absence of structural heart diseases, the risk of sudden death is low. Pharmacotherapy is commonly used as the first-line management, and catheter ablation should be considered in drug refractory ventricular arrhythmias with the purpose to improve patients’ symptoms, reduce VA burden, and preserve the left ventricular function.^9^

In patient with structural heart diseases, ventricular tachycardia is associated with increased risk of sudden cardiac death; pharmacological therapy often has limited efficacy, and implantable cardiac defibrillator (ICD) have been established as the treatment in preventing sudden death. However, in the circumstance of high burden of ventricular tachycardia, resulting in multiple ICD shocks or antitachycardia pacing despite pharmacotherapy, catheter ablation is required.^9^

With regard to ablation techniques, it has become an agreement that both endocardial and epicardial approaches may be required for effective treatment of VAs in patients with or without structural heart diseases.^7^ Because of the procedural complexity, catheter ablation for VAs is adopted primarily in high-volume centers and experienced operators. Especially when combined epicardial mapping and ablation is performed, the procedure is associated with increased risk of some important complications. This leads to the consensus that cardiothoracic surgery backup should be onsite for all patients scheduled for epicardial ablation.^8^

Ventricular ablation related complications include access-site vascular injury, cardiac perforation, tamponade, bleeding, thromboembolism or even death. Some complications are specifically associated with epicardial access, i.e., phrenic nerve injury, coronary artery damage, liver laceration or hemotoma, cardiac damage, and hemopericardium or tamponade. Table 4 summarizes the possible complications during endo/epicarial ablation and the corresponding preventive/management strategies.

**Table 4:** Possible complications during endo/epicarial access and preventive/management strategies.

Possible complications during endo/epicarial access	Preventive/management approaches
Access-site vascular injury	Careful puncture technique, appropriate image technique, post-procedural care, vascular intervention if necessary.
Thromboembolism	Pre-procedural image assessment, peri-procedural anticoagulation strategy, careful catheter/sheath flush.
Phrenic nerve injury	Phrenic nerve pacing/mapping/monitoring, balloon interposition.
Coronary artery injury	Coronary artery angiogram, balloon interposition, at least 5 mm away from the coronary artery if ablation needed.
Pericardial effusion, hemopericardium or tamponade	Pericardial drainage.
Major hemopericardium or tamponade	Pericardial drainage, blood re-circulation, cardiac surgery repair.
Major bleeding	Origin of the bleeding, (blood) transfusion, surgical repair.
Pericarditis	Systemic or intrapericardialSteroids if necessary.
Esophageal injury	Esophageal temperature monitoring if necessary.

In a prior European multicenter study involving patients undergoing epicardial ablation, major complications were observed in 4.1% patients. Eight (3.7%) patients developed tamponade, and two of them required elective surgical intervention despite pericardial drainage.^6^ In reviewing of most recent literatures, the complications rates associated with epicardial access varied from 4–13%, the main indication to surgical intervention was major pericardial bleeding, notably, no urgent surgical intervention was required.^4,10–13^

In the present study, our data represents the procedural outcome from an experienced ablation center using epicardial mapping/ablation approach in treating patients with VAs. A systematic protocol for epicardial procedure was adopted, the overall complication rate was 9.1% (4 patients), and all were hemopericardium. Three of the four patients were well managed by pericardial drainage, and one of the four patients required elective surgical repair. By carful implementing the procedural protocol, no other complication occurred; importantly no urgent surgical intervention was needed. These results may highlight the importance of (1) epicardial procedural protocol, (2) experience of the operators, (3) techniques obtaining epicardial access and catheter manipulation, (4) management strategies prepared for possible complications.^14,15^ Moreover, our data suggested that patients age was a significant risk factor for epicardial procedural complications, for patients with elder age, more attention should be paid to avoid procedural complication. Nonetheless, the current study represented a retrospective, nonrandomized, small sample-sized, single center experience.

## Conclusion

In experienced centers, guided by a tailored procedural protocol, epicardial mapping and ablation can be achieved with acceptably low complications rate. The majority of the epicardial access related complications was preventable, and can be treated conservatively without needing urgent surgical intervention. Patients with older age are associated with significantly increased risk of epicardial procedural complication.

## Disclosure / Conflict of interest

None to declare regarding the content of this study.

## Funding

None.
